# Spirometry in Greenland: a cross-sectional study on patients treated with medication targeting obstructive pulmonary disease

**DOI:** 10.3402/ijch.v75.33258

**Published:** 2016-12-08

**Authors:** Lasse Overballe Nielsen, Sequssuna Olsen, Dorte Ejg Jarbøl, Michael Lynge Pedersen

**Affiliations:** 1 Queen Ingrid Primary Health Care Center, Nuuk, Greenland; 2 Department of Pulmonology, Nordland Hospital, Bodø, Norway; 3 Research Unit of General Practice, Department of Public Health, University of Southern Denmark, Odense, Denmark; 4 Greenland Center for Health Research, Institute of Nursing and Health Science, University of Greenland, Nuuk, Greenland

**Keywords:** spirometry, COPD, Greenland, cross-sectional study, primary, practice

## Abstract

**Background:**

Chronic obstructive pulmonary disease (COPD) is globally increasing in frequency and is expected to be the third largest cause of death by 2020. Smoking is the main risk factor of developing COPD. In Greenland, more than half of the adult population are daily smokers, and COPD may be common. International guidelines recommend the usage of spirometry as a golden standard for diagnosing COPD. The current number of spirometries performed among patients treated with medication targeting obstructive pulmonary disease in Greenland remains unexplored.

**Objective:**

To estimate the prevalence of patients aged 50 years or above treated with medication targeting obstructive pulmonary disease and the extent to which spirometry was performed among them within 2 years.

**Design:**

An observational, cross-sectional study based on the review of data obtained from electronic medical records in Greenland was performed. The inclusion criterion was that patients must have been permanent residents aged 50 years or above who had medication targeting obstructive pulmonary disease prescribed within a period of 15 months prior to data extraction. A full review of electronic patient records was done on each of the identified users of medication targeting obstructive pulmonary disease. Information on age, gender, town and spirometry was registered for each patient within the period from October 2013 to October 2015.

**Results:**

The prevalence of patients treated with medication targeting obstructive pulmonary disease aged 50 years or above was 7.9%. Of those, 34.8% had spirometry performed within 2 years and 50% had a forced expiratory volume (1 sec)/ forced vital capacity (FEV1/FVC) under 70% indicating obstructive pulmonary disease.

**Conclusion:**

The use of medication targeting obstructive pulmonary disease among patients over 50 years old is common in Greenland. About one third of the patients had a spirometry performed within 2 years. To further increase spirometry performance, it is recommended to explore possible barriers in health care professionals’ usage of spirometry in different health care settings in Greenland.

Chronic obstructive pulmonary disease (COPD) is globally increasing in frequency and is expected to be the third largest cause of death by 2020 (1). COPD is frequent among smokers and former smokers, and the prevalence increases with age. Smoking is the main risk factor of developing COPD in the western world (1). In Greenland, more than half of the adult population are daily smokers (2,3). Thus, COPD may be common in Greenland too. However, the actual prevalence remains unknown (4).

International guidelines recommend the usage of spirometry as a golden standard for diagnosing COPD (1). To distinguish between the symptoms of COPD and other diseases and illnesses with similar symptoms, all adults being treated with medication targeting obstructive pulmonary disease should have a spirometry performed (1). Global Initiative for Chronic Obstructive Lung Disease (GOLD) guidelines for COPD treatment recommend an annual spirometry test for COPD patients (5). In Denmark, the aim is that 90% of all COPD patients have had an annual spirometry performed (6). The US COPD surveillance concluded that COPD is a significant source of morbidity and mortality and that the prevalence of COPD patients may be underestimated due to the limited use of spirometry in diagnosis. Furthermore, the study recommends additional efforts in education of physicians in management of COPD (7). Several studies have demonstrated that the recommended usage of spirometry and actual clinical practice are not always in coherence (8–10). However, other studies found that the use of spirometry can be improved in clinical practice with focus on training (9,11), and a Canadian study has shown that it is feasible to implement a screening program using screening questions and spirometry to identify COPD patients in primary care (12).

The health care system in Greenland aims to provide equal access to health care services for all residents. The health care system faces challenges in the geographical distances, infrastructural barriers that complicate travels between towns and settlements, high turnover of health care professionals and high financial expenditures on acute evacuations of patients (13,14). A way of ensuring coherent patient pathways might be telemedicine, which has already been used in specific specialties such as telepsychiatry, teleophthalmology, teleradiology and teledermatology (15,16). In 2008, the telemedicine store-and-forward solution (named Pipaluk) was implemented in all towns and settlements with a population of at least 50 people. Using Pipaluk, it is possible to perform several kinds of clinical testing, including spirometry (17). However, the effects of using telemedicine in COPD care remain unknown (18,19).

The health care system in Greenland initiated the lifestyle project focusing on general prevention and quality of care among patients with diabetes, hypertension and COPD (2). In 2011, a study on performed spirometries among patients treated with medication targeting obstructive pulmonary disease found a limited use of spirometry in Greenland. It was concluded that limited use of spirometry in the health care system represents a major challenge in managing COPD. The study suggested an increased focus on spirometry, including diagnosis, treatment and monitoring of COPD by providing national guidelines for documentation on electronic drug prescriptions, smoking status and spirometry testing annually (4).

As a consequence of the 2011 study (4), the lifestyle project increased focus on spirometry. All major health care centres were equipped with spirometers that could be used on existing computers identical to the spirometers already used in the Pipaluk solution. This meant that spirometry could be performed in all towns and settlements with a population of 50 people or above using the same spirometer and electronic program. In addition, a national guideline for COPD and spirometry performance was developed, including electronic documentation on prescriptions, smoking status, annual spirometry and other lifestyle factors. Teaching nurses and other health care professionals in Greenland regarding how to use spirometry was incorporated in an annual lifestyle concept course. However, the current number of spirometries performed among patients treated with medication targeting obstructive pulmonary disease remains unexplored, and the effects of the implementation of telemedicine and the initiatives by the lifestyle group are unknown.

The aim of this study was to estimate the prevalence of patients aged 50 years or above treated with medication targeting obstructive pulmonary disease and the extent to which spirometry was performed among them.

## Material and methods

### Study design

An observational, cross-sectional study based on review of data obtained from electronic medical records (EMRs) in Greenland from 1 October 2013 to 1 October 2015 was employed.

### Setting

Greenland is an island with a population of approximately 56,000 people, where most people live in the 17 towns with only around 7,500 people living in the 56 settlements (20). The majority of the Greenlandic population is of Inuit descent. The health care system is divided into five health regions, with a regional hospital in the largest town. Health care, including medication, is free of charge for all permanent residents in Greenland. One-third of the population in Greenland live in Nuuk, the capital city, where Queen Ingrid Hospital is located. This hospital functions as a central hospital for all of Greenland and is the only hospital providing secondary specialized health care (15). Since 2007, all primary health care centres and settlement consultations have used the same EMR. In 2014, the implementation of a new national EMR, also including the secondary health care at Queen Ingrid Hospital, was initiated.

Patients with COPD are most often seen at their local primary health care centre, but patients with severe COPD may be referred to and seen by visiting consultants in Internal Medicine or referred to Queen Ingrid Hospital.

### Study population and variables

The study included patients from the six largest towns of Greenland, representing 68.1% (35,818/55,984) of the entire population as of 1 January 2015 (20). The inclusion criteria were as follows: patients must have been permanent residents aged 50 years or above who had medication targeting obstructive pulmonary disease prescribed within a period of 15 months prior to data extraction. Data were extracted from the EMR in both primary and secondary health care in October 2015.

Medication targeting obstructive pulmonary disease was defined as medications with Anatomical Therapeutic Chemical code R03 (21). R03 medication includes both short- and long-acting bronchodilators as well as inhaled steroids along with other medication types for obstructive airway diseases. A full review of electronic patient records was done for each of the identified users of medication targeting obstructive pulmonary disease.

Information on age, gender and spirometry was registered for each patient. Information on forced expiratory volume (1 sec) (FEV1), forced vital capacity (FVC) and FEV1/FVC was registered. An FEV1/FVC value under 70% was considered as pulmonary obstruction. Only the most recent spirometry test was included and only the best test if more than one test was performed on the same day on the same patient. The spirometer used in the selected towns was Midmark IQspiro^®^ (Midmark Diagnostics Group, Gardena, CA, USA).

### Statistical analysis

The prevalence of patients aged 50 years or above being treated with medication targeting obstructive pulmonary disease was estimated using the population of Greenland as of 1 January 2015 as background population (20). The number of performed spirometries within the 2-year period was recorded with values of FEV1, FVC and FEV1/FVC prior to data extraction. The chi-square test was used to compare frequencies between gender and towns, respectively. Estimates were calculated with 95% confidence intervals (CI). A *p*-value under 0.05 was used as the level of significance. All analyses were performed using SPSS, Version 23 (IBM SPSS Statistics). The study was approved by the Ethics Committee for Medical Research in Greenland (No. 2015-23) and the Agency of Health and Prevention in Greenland.

## Results

In total, 782 patients aged 50 years or above were identified as having received an electronic prescription for medication targeting obstructive pulmonary disease in the 15-month period from July 2014 to October 2015. The mean age of the patients was 64.1 (SD±9.5); there was no statistical significant difference between genders (see Table I); however, the mean age was significantly different between Nuuk and Towns, *p*=0.001 (see Table II). The prevalence of patients aged 50 or above treated with medication targeting obstructive pulmonary disease was 7.9% (95% CI: 7.4–8.5%) (see Table I). The prevalence of medication use was significantly higher among women (11.0%) than among men (5.4%), *p*<0.001 (see Table I). Between Nuuk and the five towns, there was no significant difference in prevalence of patients aged 50 or above treated with medication targeting obstructive pulmonary disease (*p*≤0.812; see Table II).

**Table I:** Prevalence of medication and proportion of spirometry performed in patients aged 50 years or above (gender)

Variables	Total% (95% CI) (n/N)	Women% (95% CI) (n/N)	Men% (95% CI) (n/N)	p
Mean age (SD)	64.1 (±9.5)	63.7 (±9.7)	64.6 (±9.1)	0.224
Prevalence of medication use within a 15-month interval	7.9 (7.4–8.5%) (782/9,853)	11.0 (10.1–11.9%) (492/4,483)	5.4 (4.8–6.0%) (290/5,370)	<0.001
Spirometry performed within 2 years	34.8 (31.4–38.2%) (272/782)	33.3 (39.2–37.5%) (164/492)	37.2 (31.7–42.8%) (108/290)	0.268
FEV1/FVC under 70%	50.0 (44.1–55.9%) (136/272)	41.5 (33.9–49.0%) (68/164)	63.0 (53.9–72.1%) (68/108)	<0.001

FEV1, forced expiratory volume (1 sec); FVC, forced vital capacity.

**Table II:** Prevalence of medication and proportion of spirometry performed in patients aged 50 years or above (Nuuk/towns)

Variables	Total% (95% CI) (n/N)	Nuuk% (95% CI) (n/N)	Towns% (95% CI) (n/N)	p
Mean age (SD)	64.1 (±9.5)	62.8 (±9.0)	65.1 (±9.7)	0.001
Prevalence of medication use within a 15-month interval	7.9 (7.4–8.5%) (782/9,853)	7.9 (7.1–8.7%) (350/4,450)	8.0 (7.3–8.7%) (432/5,403)	0.812
Spirometry performed within 2 years	34.8 (31.4–38.2%) (272/782)	36.0 (31.0–41.0%) (126/350)	33.8 (29.3–38.3%) (146/432)	0.520
FEV1/FVC under 70%	50.0 (44.1–55.9%) (136/272)	46.8 (38.1–55.5%) (59/126)	52.7 (44.6–60.8%) (77/146)	0.331

FEV1, forced expiratory volume (1 sec); FVC, forced vital capacity.

Spirometry was performed in 34.8% (95% CI: 31.4–38.1%) of patients aged 50 or above and treated with medication targeting obstructive pulmonary disease. No statistical significant difference between genders (see Table I) or between Nuuk and towns (see Table II) was observed. The proportion of patients with an FEV1/FCV value under 70% was 50% (95% CI: 44.1–55.9%). A significantly higher proportion of males (63.0%, 95% CI: 53.9–72.1%) had an FEV1/FVC under 70% (p <0.001; see Table I). The proportion of patients with an FEV1/FCV value under 70% was not statistically significant when comparing Nuuk with the five towns.

The proportions of patients tested with spirometry within 2 years differed significantly among towns, ranging from 22.0 to 49.0% (*p*=0.008). There was no statistically significant trend between age progression and FEV1/FVC under 70% (*p*=0.505). This was also the case between genders: men, *p*=0.474; women, *p*=0.945.

## Discussion

The prevalence of patients aged 50 years or above treated with medication targeting obstructive pulmonary disease was 7.9%. About one-third of patients had a spirometry performed within 2 years, and half of the patients had an FEV1/FVC under 70% indicating obstructive pulmonary disease.

The major strength of this study was that 68% of the entire Greenlandic population was covered and patients were identified electronically. Still, the number of patients was limited, and results should be taken with reservations. Also, some prescriptions may have been done non-electronically, leading to an underestimation of the number of patients included. However, the use of electronic prescriptions is standard and the easiest way to prescribe medicine properly, which limits the risk of underestimation. On the contrary, the estimate of treated patients may be overestimated since no information about compliance was available. Yet, with free medication and pharmacy localized at the health care clinics, the extent of the overestimation is considered minimal. Similarly, it is possible that not all performed spirometries were registered in the EMR, leading to an underestimation of the included spirometries. However, all performed clinical tests must be documented by law, and a possible underestimation is considered insignificant. It was not possible to distinguish between COPD and asthma diagnosis in the study; however, spirometry testing is indicated with all patients above 50 years in treatment with medication targeting obstructive pulmonary disease (5,22).

The prevalence of patients treated with medication targeting obstructive pulmonary disease in this study represents an increase of 25% compared to the prevalence of 6.3% reported in 2011 (4). This may reflect an increase of prescriptions on medication targeting obstructive pulmonary disease or an improvement in using electronic documentation. Also, the natural prevalence of obstructive pulmonary diseases may have increased. The number of people 65+ increased by 10% since 2011, which could contribute to the rise of patients treated with medication targeting obstructive pulmonary disease (20). More women than men were treated with medication targeting obstructive pulmonary disease, which may reflect that women in Greenland have more frequent contact to the primary health care system (23). Similar results have been found in other Nordic countries (24–26).

The proportion of patients with a performed spirometry within 2 years in this study represents more than a double of the proportion of 14.1% reported in 2011 (4), indicating an increased awareness of spirometry within the Greenlandic health care system. This could suggest an effect of the initiatives by the lifestyle project.

This is in accordance with a study on spirometry performance in primary health care from Denmark, which reported that an educational program based on GOLD guidelines could increase spirometry performance (11). However, the proportion of patients tested with spirometry in the present study is still lower than that of the Denmark study. Of the patients with prescribed medication targeting obstructive pulmonary disease for the first time in 2008 in a primary health care setting in Denmark, about 50% had a spirometry performed (27). This study found more spirometry performed in partnership practices compared to single-handed practices and also among younger doctors compared to older doctors (27), indicating that the size of the primary clinic and the number of doctors have an impact on the number of performed spirometries. In our study, the difference among the towns was observed underlining the importance of a primary health care setting, which suggested room for improvement in some towns. On the other hand, the limited usage of spirometry in Greenland is not unique. A recent survey including 12 countries across the Asia-Pacific region, Africa, Eastern Europe and Latin America found that the usage of spirometry when diagnosing COPD is underutilized as the median of performed spirometries across the countries was 26%, which proves that spirometry performance is a global challenge (28).

Only one-third of the patients treated with medication targeting obstructive pulmonary disease had a spirometry performed within 2 years. The reluctance to perform spirometry is problematic in a population with a prevalence of 50% daily smokers, and COPD is most likely underdiagnosed in Greenland (2,3). Studies from other circumpolar areas in North America have shown similar smoking percentages among the aboriginal people (29–32). Among the Inuit people in Canada, the prevalence of diagnosed COPD was 10.1% (29), and the estimated worldwide prevalence is between 5 and 10% in people aged 40 years and above (33). Thus, increased awareness of screening for COPD among adult smokers in Greenland seems indicated as well as more public awareness of the consequences of smoking and specific smoking cessation programmes adapted to the Greenlandic culture (34).

In conclusion, the use of medication targeting obstructive pulmonary disease among patients over 50 years old is common. Despite increased awareness of COPD in the health care system in Greenland, including national standardized spirometry equipment in all health care settings, national guidelines, educational initiatives and written and visual patient information, only one-third of the patients had a spirometry performed within 2 years, indicating possible barriers to perform spirometry in the Greenlandic health care system. The Greenlandic health care system aims to follow international standards of COPD treatment, and in order to increase spirometry performance, it is recommended to further explore health care professionals’ usage of spirometry in different health care settings in Greenland.

## References

[1] (2015). From the Global Strategy for the Diagnosis, Management and Prevention of COPD. http://www.goldcopd.org/.

[2] Reiss AE, Pedersen ML (2014). Smoking among patients in the primary health care system in Nuuk, Greenland. Clin Nurs Stud.

[3] Dahl-Petersen IK, Larsen CVL, Nielsen NO, Jørgensen ME, Bjerregaard P (2016). Befolkningsundersøgelsen i Grønland 2014. Levevilkår, livsstil og helbred.

[4] Olsen S, Jarbøl DE, Kofoed M, Abildskov K, Pedersen ML (2013). Prevalence and management of patients using medication targeting obstructive lung disease: a cross-sectional study in primary healthcare in Greenland. Int J Circumpolar Health.

[5] Global Initiative for Chronic Obstructive Lung Disease (GOLD) http://www.goldcopd.org/guidelines-global-strategy-for-diagnosis-management.html.

[6] Dansk register for Kronisk Obstruktiv Lungesygdom National Årsrapport 2011.

[7] Ford ES, Croft JB, Mannino DM, Wheaton AG, Zhang X, Giles WH (2013). COPD surveillance–United States, 1999–2011. Chest.

[8] Arne M, Lisspers K, Ställberg B, Boman G, Hedenström H, Janson C (2010). How often is diagnosis of COPD confirmed with spirometry?. Respir Med.

[9] Buffels J, Degryse J, Heyrman J, Decramer M, DIDASCO Study (2004). Office spirometry significantly improves early detection of COPD in general practice: the DIDASCO Study. Chest.

[10] Koefoed MM, dePont Christensen R, Søndergaard J, Jarbøl DE (2012). Lack of spirometry use in Danish patients initiating medication targeting obstructive lung disease. Respir Med.

[11] Lange P, Rasmussen FV, Borgeskov H, Dollerup J, Jensen MS, Roslind K (2007). The quality of COPD care in general practice in Denmark: the KVASIMODO study. Prim Care Respir J.

[12] Lee L, Patel T, Hillier LM, Milligan J (2016). Office-based case finding for chronic obstructive pulmonary disease in older adults in primary care. Can Respir J.

[13] Nelson C (2013). The polar bear in the room: diseases of poverty in the Arctic. Int J Circumpolar Health.

[14] Young TK, Chatwood S (2011). Health care in the North: what Canada can learn from its circumpolar neighbours. CMAJ.

[15] Højgaard H (2016). Professional challenges both attract and deter. https://dsr.dk/sygeplejersken/arkiv/sy-nr-2016-2/faglige-udfordringer-baade-tiltraekker-og-skraemmer.

[16] Højgaard H With telemedicine a friend is always around the corner. Tikiusaaq, 24.

[17] Højgaard H (2016). Telemedicine promotes equitable access to healthcare ∣ Sygeplejersken, DSR. https://dsr.dk/sygeplejersken/arkiv/sy-nr-2016-2/telemedicin-medvirker-til-lige-adgang-til-sundhedsydelser.

[18] Ure J, Pinnock H, Hanley J, Kidd G, McCall Smith E, Tarling A (2012). Piloting tele-monitoring in COPD: a mixed methods exploration of issues in design and implementation. Prim Care Respir J.

[19] Pinnock H, Hanley J, McCloughan L, Todd A, Krishan A, Lewis S (2013). Effectiveness of telemonitoring integrated into existing clinical services on hospital admission for exacerbation of chronic obstructive pulmonary disease: researcher blind, multicentre, randomised controlled trial. BMJ.

[20] Statistics Greenland http://www.stat.gl/.

[21] WHOCC ATC/DDD index. http://www.whocc.no/atc_ddd_index/.

[22] Bateman ED, Hurd SS, Barnes PJ, Bousquet J, Drazen JM, FitzGerald M (2008). Global strategy for asthma management and prevention: GINA executive summary. Eur Respir J.

[23] Pedersen ML, Rolskov A, Jacobsen JL, Lynge AR (2012). Frequent use of primary health care service in Greenland: an opportunity for undiagnosed disease case-finding. Int J Circumpolar Health.

[24] Vehviläinen AT, Kumpusalo EA, Voutilainen SO, Takala JK (1995). General practice consultations in central and northern Finland. Scand J Prim Health Care.

[25] Eggen P, Maeland JG, Skjaerven R (1993). Use of primary medical care: does place of residence play a role?. Scand J Prim Health Care.

[26] Njálsson T (1995). On content of practice. The advantage of computerized information systems in family practice. Scand J Prim Health Care.

[27] Koefoed MM, Søndergaard J, Christensen Rd, Jarbøl DE (2013). General practice variation in spirometry testing among patients receiving first-time prescriptions for medication targeting obstructive lung disease in Denmark: a population-based observational study. BMC Fam Pract.

[28] Aisanov Z, Bai C, Bauerle O, Colodenco FD, Feldman C, Hashimoto S (2012). Primary care physician perceptions on the diagnosis and management of chronic obstructive pulmonary disease in diverse regions of the world. Int J Chron Obstruct Pulmon Dis.

[29] Ospina MB, Voaklander D, Senthilselvan A, Stickland MK, King M, Harris AW (2015). Incidence and prevalence of chronic obstructive pulmonary disease among aboriginal peoples in Alberta, Canada. PLoS One.

[30] Egeland GM, Cao Z, Young TK (2011). Hypertriglyceridemic-waist phenotype and glucose intolerance among Canadian Inuit: the International Polar Year Inuit Health Survey for Adults 2007–2008. CMAJ.

[31] Chateau-Degat M-L, Dewailly É, Louchini R, Counil É, Noël M, Ferland A (2010). Cardiovascular burden and related risk factors among Nunavik (Quebec) Inuit: insights from baseline findings in the circumpolar Inuit health in transition cohort study. Can J Cardiol.

[32] Dilley JA, Peterson E, Bobo M, Pickle KE, Rohde K (2013). Tobacco use prevalence – disentangling associations between Alaska Native race, low socio-economic status and rural disparities. Int J Circumpolar Health.

[33] Halbert RJ, Natoli JL, Gano A, Badamgarav E, Buist AS, Mannino DM (2006). Global burden of COPD: systematic review and meta-analysis. Eur Respir J.

[34] Jensen AB, Hounsgaard L (2013). “I only smoke when I have nothing to do”: a qualitative study on how smoking is part of everyday life in a Greenlandic village. Int J Circumpolar Health.

